# High-selective HDAC6 inhibitor alleviates bone marrow fibrosis through inhibiting collagen formation and extracellular matrix deposition

**DOI:** 10.1038/s41598-025-08384-6

**Published:** 2025-08-01

**Authors:** Chiao-Hsu Ke, Mao-En Huang, Hsin-Yi Wu, Chao-Wu Yu, Shuei-Liong Lin, Shau-Ping Lin, Shu-Han Yu, Chih-Hung Huang, Chen-Si Lin

**Affiliations:** 1 Department of Veterinary Medicine, School of Veterinary Medicine, National Taiwan University, No. 1, Sec. 4, Roosevelt Rd., Taipei, 10617 Taiwan, ROC; 2 Instrumentation Center, National Taiwan University, Taipei, 10617 Taiwan; 3 School of Pharmacy, College of Medicine, National Taiwan University, Taipei, Taiwan; 4 Graduate Institute of Physiology, College of Medicine, National Taiwan University, Taipei, 100 Taiwan; 5 Department of Internal Medicine, Department of Integrated Diagnostics and Therapeutics, National Taiwan University Hospital, Taipei, 100 Taiwan; 6 Agricultural Biotechnology Research Center, Academia Sinica, Taipei, 115 Taiwan; 7 Center of Systems Biology, The Research Center of Developmental Biology and Regenerative Medicine, National Taiwan University, Taipei, 106 Taiwan; 8 Institute of Biotechnology, National Taiwan University, Taipei, 106 Taiwan; 9 Department of Chemical Engineering and Biotechnology, Institute of Chemical Engineering, National Taipei University of Technology, Taipei, 10608 Taiwan

**Keywords:** Bone marrow fibrosis, Histone deacetylase inhibitor, Transforming growth factor-β (TGF-β), Organ fibrosis, Cell biology, Drug discovery, Molecular biology, Diseases, Molecular medicine

## Abstract

Bone marrow fibrosis (BMF) impairs normal hematopoietic functions in patients. The overactivation of the TGF-β signaling pathway is regarded as one of the offenders causing disease progression. Thus, factors capable of regulating TGF-β secretion hold great potential in reversing fibrotic diseases. One such factor is histone deacetylase inhibitors (HDACis), which can modulate the expression of TGF-β. Our previous study successfully synthesized a selective HDAC6 inhibitor, J22352, for pulmonary fibrosis; however, the treatment efficacies on BMF remain unclear. Therefore, in this study, we treated bone marrow-derived myofibroblasts with J22352. The results showed that J22352 significantly reduced cell viability, induced apoptosis, and inhibited extracellular matrix (ECM) accumulation. Liquid chromatography-tandem mass spectrometry (LC-MS/MS) analysis was employed to disclose potential mechanisms, identifying 334 differentially expressed proteins (DEPs). The DEPs were involved in cell apoptosis, programmed cell death, ECM deposition, and collagen formation. These results suggest that J22352 efficiently alleviated BMF by inducing cell apoptosis and inhibiting ECM deposition. This study introduces a novel selective HDAC6 inhibitor as a potential option for slowing down the progression of BMF. We aim to provide a promising selective HDACi for clinical medicine through a detailed analysis of its mechanisms and efficacy, offering new prospects in the field.

**Supplementary Information:**

The online version contains supplementary material available at 10.1038/s41598-025-08384-6.

## Introduction

Fibrosis is a physiologically detrimental tissue-repair mechanism in humans that can lead to end-stage, multiorgan failure. When the body undergoes injury, the matrix biosynthesis machinery responds by initiating the repair of the damaged tissue and subsequently ensuring that homeostasis is retained^1^. Bone marrow fibrosis (BMF) is characterized by the increased deposition of reticulin fibers and collagen fibers^2^. Fibrosis in bone marrow impairs normal hematopoietic functions. Several hematologic and non-hematologic disorders are highly associated with BMF^3^. The pathogenesis of BMF is poorly understood. In such patients, megakaryocytes aberrantly proliferate and exhibit clustering in the bone marrow^4^ thereby leading to the increased emperipolesis of neutrophils^5^. Then the neutrophils abnormally release their enzymes in the megakaryocytes, which leads to the increased production of cytokines such as transforming growth factor-β (TGF-β), platelet-derived growth factor (PDGF), and fibroblast growth factor (FGF)^5^. These growth factors stimulate fibroblasts to generate fibrosis and endothelial cells to cause neoangiogenesis^6^. Among these cytokines, TGF-β is a leading cytokine that stimulates fibroblasts to produce extracellular matrix (ECM)^2^. Furthermore, in Gata-1^low^ mice (elevated TGF-β1 levels), a well-known murine model for BMF, the TGF-β1 content in megakaryocytes was increased by 5- to 10-fold^7^. Therefore, BMF is thought to be a consequence of the overproduction of TGF-β from megakaryocytes, leading to fibrosis in the bone marrow^4^. However, to date, allogeneic hematopoietic stem cell transplantation is currently the only treatment option that has curative potential in these patients^8^. These difficulties highlight the urgent demand to develop effective medical interventions, which could cure and/or reduce disease severity.

The TGF-β signaling pathway is one of the offenders that manipulates the activation of fibroblasts and epithelial cells causing tissue fibrosis in various organs^9–11^. Therefore, targeting the signaling cascade downstream of TGF-β presents a new approach to mitigate organ fibrosis. Increasing evidence suggests that histone deacetylases (HDACs) play vital roles in regulating the expression of genes associated with TGF-β signaling and tissue fibrosis^12^. Therefore, pan-HDAC inhibitors such as givinostat^13^vorinostat^14^and panobinostat^15^ have been used to treat several fibrosis-related disorders, hematological malignancies, and myelofibrosis. However, pan-HDAC inhibitors usually have off-target effects, highlighting the importance of developing inhibitors of specific HDAC subtypes to fight against fibrosis^16^. HDAC6, one of the subtypes of HDAC, is a microtubule-associated deacetylase that can promote fibrosis via TGF-β1/Smad3 signaling during the epithelial-mesenchymal transition^17–19^. A previous study showed the anti-fibrotic properties of HDAC6 inhibitors, which suppressed the TGF-β/Smad and EGFR/Akt profibrotic pathways^20^. Furthermore, the expressions of collagen and Akt phosphorylation induced by TGF-β1 were relieved by HDAC6 inhibitors^21^. Therefore, targeting HDAC6 has a high potential to alleviate TGF-β-induced fibrosis.

In our previous study, we successfully synthesized an HDAC 6 inhibitor against pulmonary fibrosis^22^. However, whether this drug had the potential to be applied to BMF remains unknown. Therefore, in the current study, we examined whether the novel HDAC6 inhibitor, J22352, can induce therapeutic effects in BMF and explored mechanisms by which J22352 might regulate the expression of fibrogenic genes or proteins. We found that J22352 induced fibrotic cell apoptosis and suppressed the fibrosis-related gene expressions. In proteomic results, J22352 treatments enhanced the apoptotic process and histone modification. Our data demonstrate that J22352 alleviates BMF, supporting a new anti-fibrotic therapeutic strategy for BMF.

## Results

### TGF-β1 induced bone marrow-derived fibroblast cell proliferation through the elevated collagen formation

**Fig. 1:**
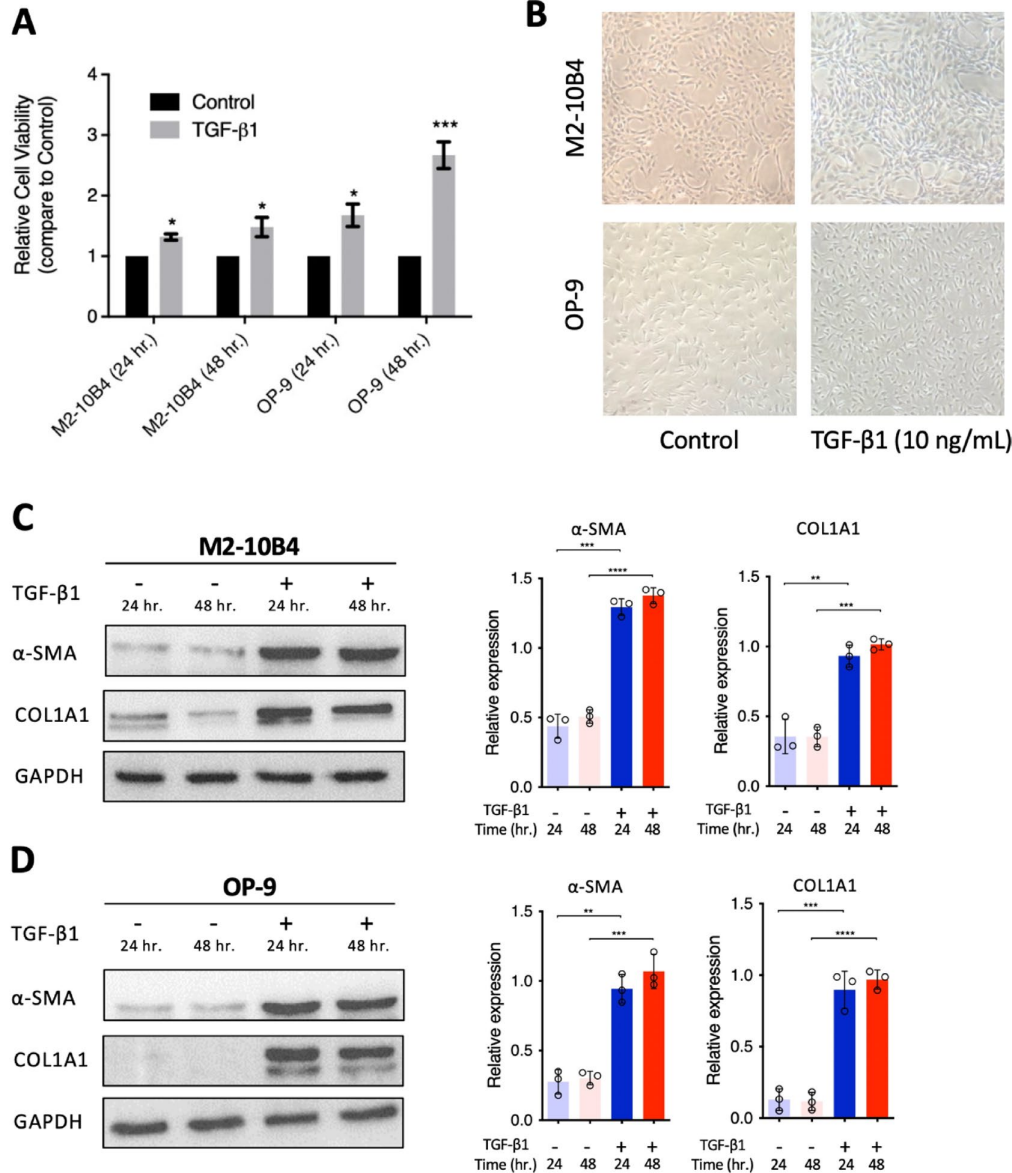
TGF-β1 mediated proliferation and induced collagen deposition in BM-derived fibroblasts. (**A**) Cell proliferation in M2-10B4 and OP-9 cells with TGF-β1 (10 ng/mL) induction. (**B**) Representative figures show the cell growth in M2-10B4 and OP-9 cells induced by the TGF-β1 stimulation. (**C**) Representative western blot images and quantification in M2-10B4 and (**D**) OP-9 cells, showing the increased levels of α-SMA and COL1A1 triggered by TGF-β1. Bar graphs reflect mean ± SEM, and data were analyzed by the Student t-test. *P < 0.05; **P < 0.01; ***P < 0.001; ****P < 0.0001.

TGF-β1 is the key cytokine that participates in different organ fibrosis, including BMF. Thus, TGF-β1 was first applied to stimulate the proliferation of two BM-derived fibroblasts, M2-10B4 and OP-9 cell lines. Compared to the vehicle control, TGF-β1 (10 ng/ml) significantly increased the cell viability of M2-10B4 to 131.8% ± 5.0% and 148.1% ± 15.5% at 24 and 48 h, respectively. Similarly, TGF-β1 (10 ng/ml) can also effectively induce the cell proliferation of OP-9 cells to 167.5% ± 19.0% and 267.0% ± 22.0% at 24 and 48 h (Fig. 1A and B). The expressions of alpha-smooth muscle actin (α-SMA) and collagen (COL1A1) significantly increased in BM-derived fibroblast after TGF-β1 induction (Fig. 1C and D). These results indicated that TGF-β1 induced BM-derived fibroblast cell proliferation through the enhancement of collagen formation.

### J22352 significantly suppressed HDAC6 activities in BM-derived fibroblasts

**Fig. 2:**
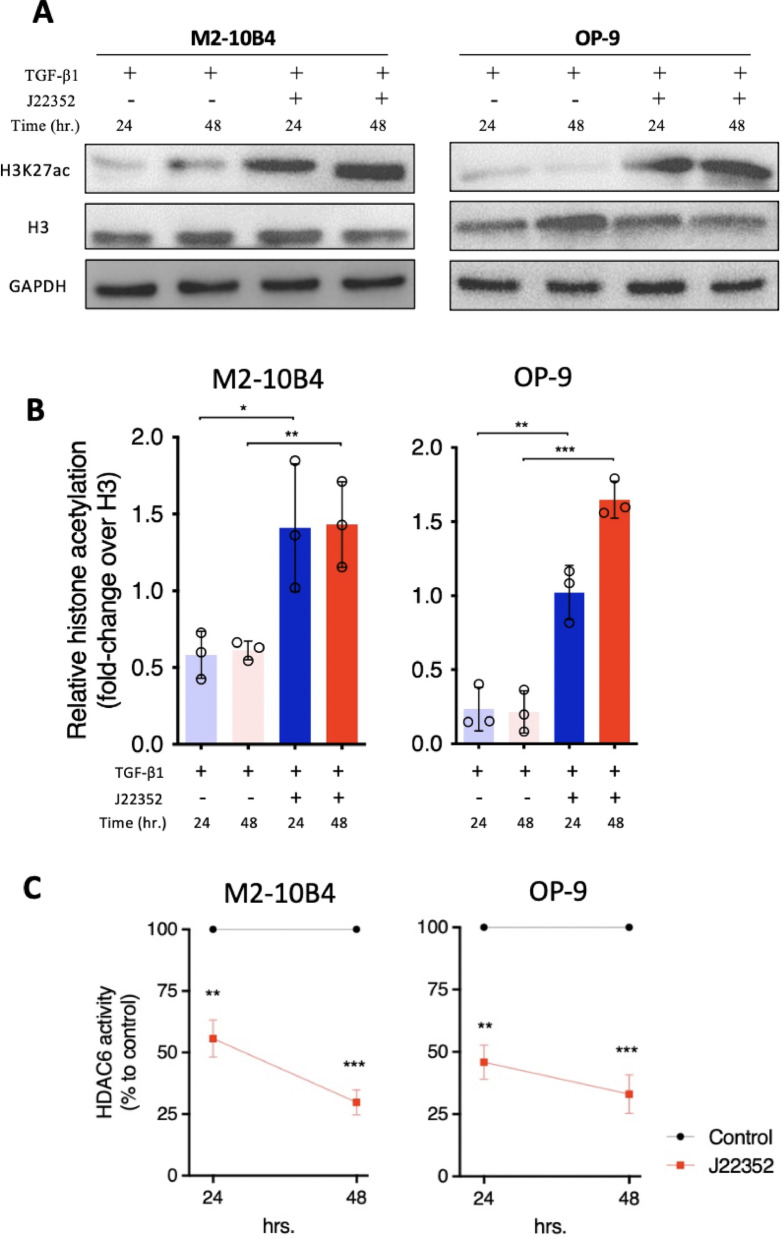
J22352 effectively increased histone acetylation and suppressed HDAC6 activity in BM-derived fibroblasts compared with non-treated control. (**A**) Representative figures of western blots. Histone modifications change following treatment with J22352 for 24 and 48 h. Nuclear extracts were subjected to immunoblots. (**B**) Densitometric analysis plots corresponding to western blot analysis. GAPDH was used as a loading control. H3K27ac values were then normalized to the H3. (**C**) Assessment of HDAC6 activity in BM-derived fibroblasts upon J22352 treatments for 24 and 48 h. Working concentrations of each cytokine and/or J22352 were described as followed. TGF-β1: 10 ng/mL; J22352: 15.83 and 5.02 µM for 24 and 48 h (M2-10B4 cells); J22352: 82.0 and 13.46 µM for 24 and 48 h (OP-9 cells). Data presented as mean ± SEM and analyzed by the Student t-test. *P < 0.05; **P < 0.01; ***P < 0.001.

Our previous study reported the deacetylation effects of J22352 in pulmonary fibrosis; however, the roles of J22352 in BMF remain unclear. After J22352 treatment for 24 and 48 h, the expressions of H3K27ac significantly increased in TGF-β1 induced BM-derived fibroblasts (Fig. 2A and B). Furthermore, the HDAC6 activity in these two cell lines was also significantly decreased (Fig. 2C). These results indicated that J22352 effectively induced histone acetylation and therefore suppressed HDAC6 activity in BM-derived fibroblasts.

### Novel HDAC 6 inhibitor induced early apoptosis in BM-derived fibroblasts

**Fig. 3:**
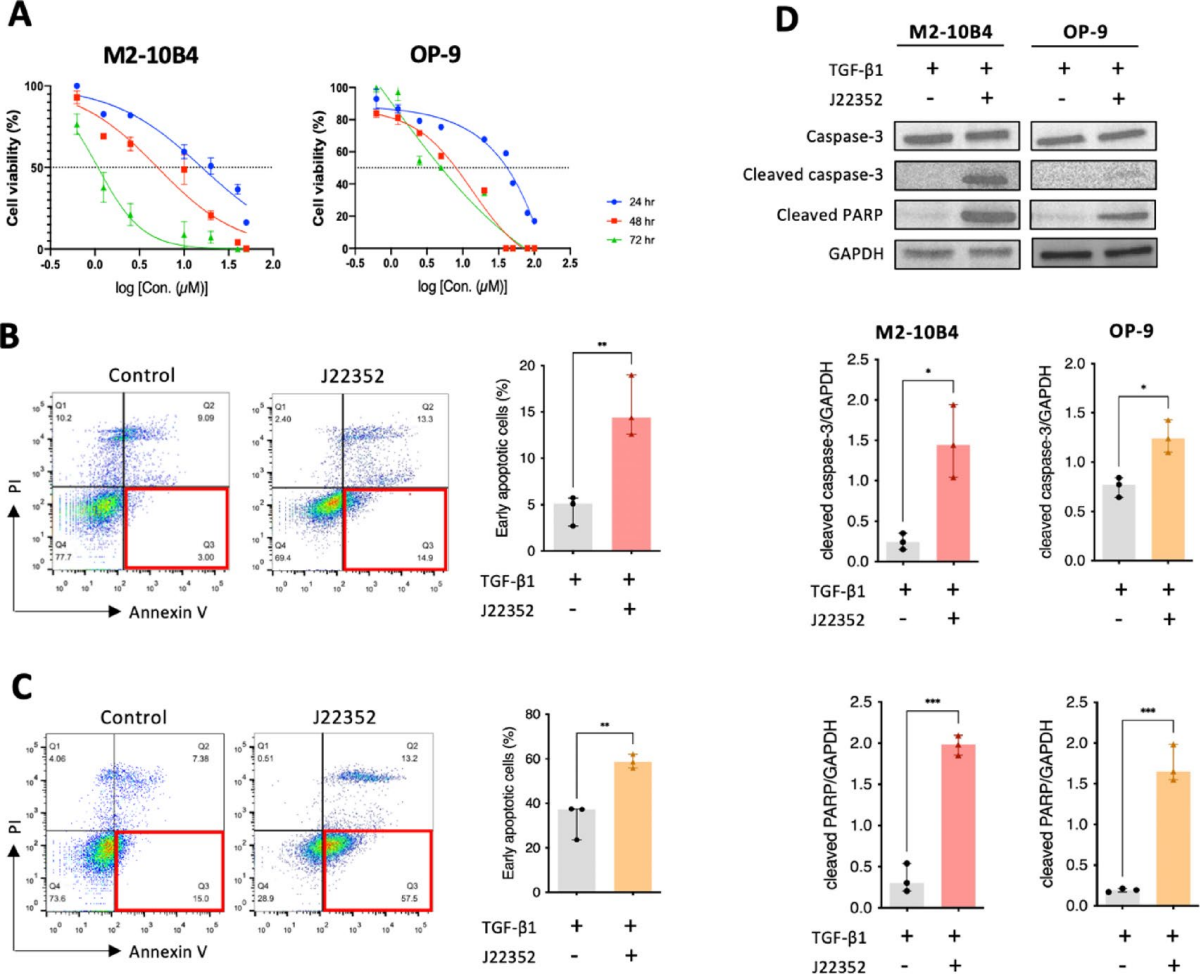
The in-house synthesized HDAC6 selective inhibitor (J22352) induced early apoptosis in BM-derived fibroblasts. (**A**) The half-maximal inhibitory concentration (IC_50_) of J22352 in M2-10B4 and OP-9 cells. (**B**) Representative flow cytometric plots of M2-10B4 and (**C**) OP-9 cells are shown. Quadrants indicate viable cells (lower left quadrant), early apoptosis (lower right quadrant), late apoptosis (upper right quadrant), and necrotic cells (upper left quadrant). (**D**) Western blot analysis of the expression of apoptosis proteins, caspase-3, cleaved caspased-3, and cleaved PARP, in M2-10B4 and OP-9 cells. Working concentrations of each cytokine and/or J22352 were described as followed. TGF-β1: 10 ng/mL; J22352: 15.83 µM for 24 h (M2-10B4 cells); J22352: 82.0 µM for 24 h (OP-9 cells).Each bar reflects the mean ± SEM of three independent experiments. Data were analyzed by the Student t-test. **P < 0.01.

To determine the cytotoxic effects of the novel in-house synthesized HDAC6 inhibitor, J22352, on BM-derived fibroblast, an assessment of the half-maximal inhibitory concentrations (IC_50_) was conducted. After J22352 treatments at various concentrations, the dose-dependent effects of J22352 on cell viability were determined, as summarized in Fig. 3A. J22352 decreased the cell numbers in these tested cell lines with different IC_50_. For M2-10B4 cells, the estimated IC_50_ were 15.83 µM, 5.02 µM, and 1.07 µM at 24, 48, and 72 h. The estimated IC_50_ of OP-9 cells were 82.0 µM, 13.46 µM, and 0.78 µM at 24, 48, and 72 h, respectively (Table 1). Therefore, in addition to the dose-dependent cytotoxic abilities, the drug effect was persistent even at 48 and 72 h, which revealed that J22352 also inhibited cell growth in a time-dependent manner.

**Table 1:** The estimated IC_50_ of J22352 in BMF cells.

BMF cells/incubation time	24 h (µM)	48 h (µM)	72 h (µM)
M2-10B4	15.83	5.02	1.07
OP-9	82.0	13.46	0.78

To investigate the potential mechanisms of J22352 on bone marrow-derived fibroblasts, flow cytometry with Annexin V/PI double staining was employed to assess apoptosis in these cells. Early apoptotic cells were classified as Annexin V+/PI-. The pattern of apoptosis varied between the control and treatment groups. The proportions of M2-10B4 cells exhibiting early apoptosis in the control and treatment groups were 4.50 ± 0.92% and 15.33 ± 1.91%, respectively (Fig. 3B, *p* < 0.01). In OP-9 cells, the percentage of early apoptotic cells was significantly increased in the J22352-treated group (58.87 ± 1.80%) compared with that in the control group (32.80 ± 4.60%, Fig. 3C, *p* < 0.01). Western blot analysis of cleaved caspase-3 and cleaved PARP revealed the ability of J22352 to induce apoptotic effects in these cells (Fig. 3D). These results indicated that HDACi induced cell early apoptosis in BM-derived fibroblasts.

### Decreased transcript levels of ECM deposition by J22352 treatments

**Fig. 4:**
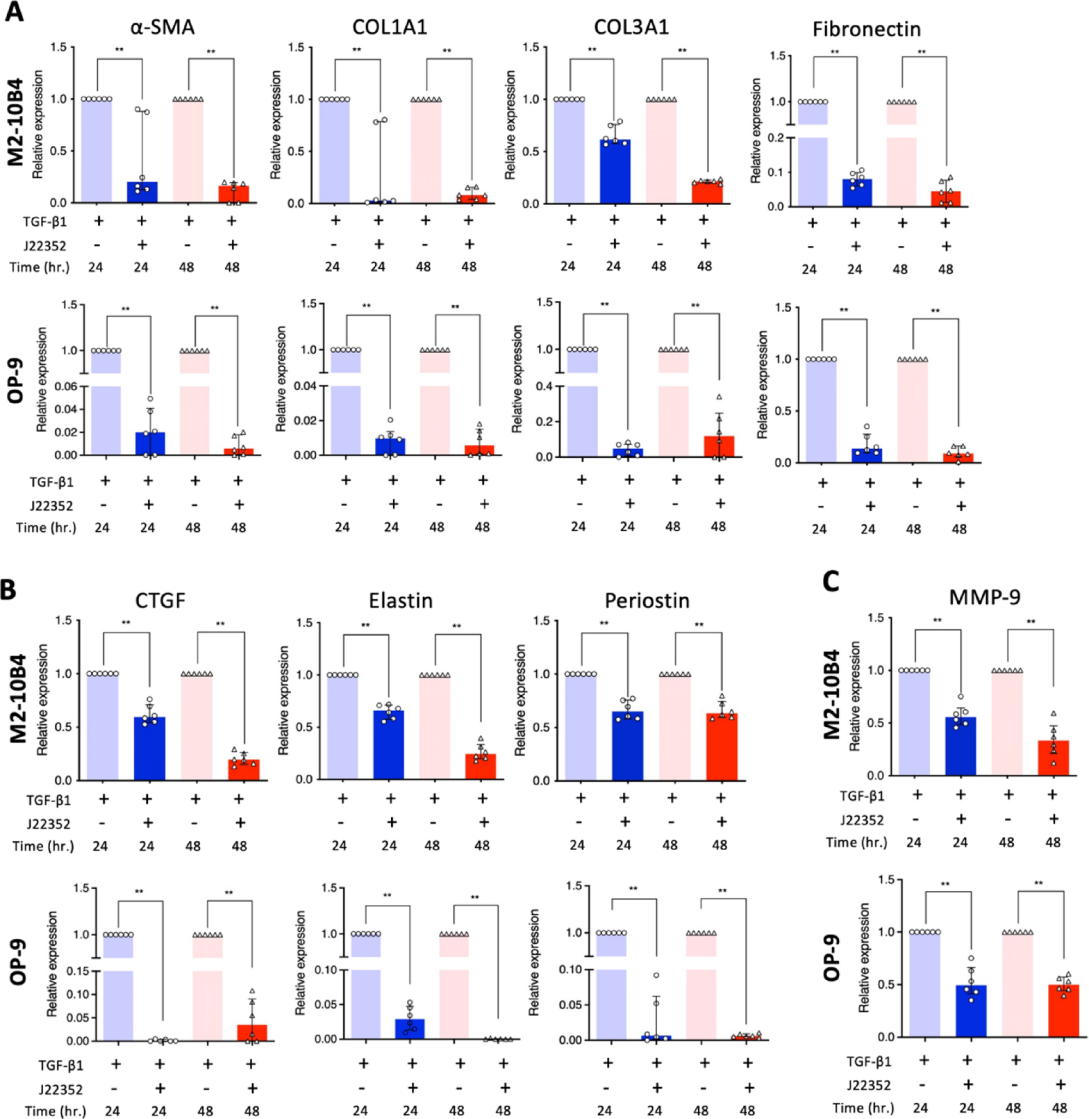
Downregulated expressions of collagen and ECM-related genes with J22352 treatments in BM-derived fibroblasts. (**A**) mRNA expressions of α-SMA, COL1A1, COL3A1, Fibronectin, (**B**) CTGF, Elastin, Periostin, and (**C**) MMP-9 in TGF-β1-induced M2-10B4 and OP-9 cells. Working concentrations of each cytokine and/or J22352 were described as followed. TGF-β1: 10 ng/mL; J22352: 15.83 and 5.02 µM for 24 and 48 h (M2-10B4 cells); J22352: 82.0 and 13.46 µM for 24 and 48 h (OP-9 cells). Data presented as median ± interquartile range and analyzed by the Mann-Whitney test. n.s., no significant difference; *P < 0.05; **P < 0.01; ***P < 0.001; ****P < 0.0001.

The aforementioned results suggested that HDAC inhibition triggered the BM-derived fibroblast apoptosis. Thus, several genes representing fibrosis were selected for further evaluation. For example, *α-SMA*, *Col1a*, *Col3a*, and *FN1* were correlated with myofibroblast activation and extracellular matrix synthesis. As shown in Fig. 4A, we observed that J22352 treatment decreased gene expression in the fibrosis formation stage at 24 and 48 h. Furthermore, some fibroblast differentiation-associated genes, *Ctgf*, *Elastin*, and *Periostin*, were also decreased after J22352 induction (Fig. 4B). Then, one of the ECM production genes, *MMP-9*, was also downregulated after treatment (Fig. 4C). These results suggested that J22352-treated fibroblasts may have lost their anti-fibrogenic ability compared to controls.

### J22352 suppresses TGF-β1/SMAD signaling pathways and HDAC6 activity

**Fig. 5:**
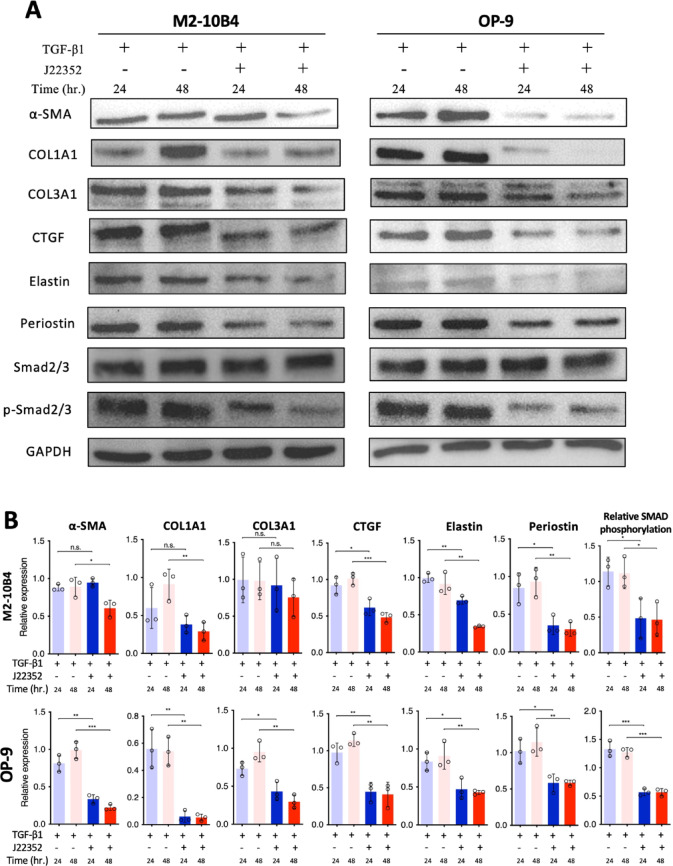
J22352 inhibited collagen formation, ECM deposition, and SMAD phosphorylation in BM-derived fibroblasts. (**A**) Representative western blot images and (**B**) quantification of α-SMA, COL1A1, COL3A1, CTGF, Elastin, Periostin, SMAD 2/3, and phosphorylated SMAD 2/3 expression in M2-10B4 and OP-9 cells. Working concentrations of each cytokine and/or J22352 were described as followed. TGF-β1: 10 ng/mL; J22352: 15.83 and 5.02 µM for 24 and 48 h (M2-10B4 cells); J22352: 82.0 and 13.46 µM for 24 and 48 h (OP-9 cells). Data presented as mean ± SEM of three independent experiments. Data were analyzed by the Student t-test. n.s., no significant difference; *, *P* < 0.05; **, *P* < 0.01; ***, *P* < 0.001.

We next investigated the anti-fibrotic effects of J22352 by the Western blot analysis. As shown in Fig. 5, J22352 significantly reduced the expression levels of α-SMA and ECM-related proteins, including COL1A1, COL3A1, CTGF, Elastin, and Periostin, in BM-derived fibroblasts. Notably, with J22352 treatment, decreased expressions of the phosphorylated Smad2/3, key proteins in the TGF-β signaling pathway, were found. These results suggested that J22352 reduced fibrotic proteins and inhibited the TGF-β signaling pathway, thereby attenuating fibrosis in BM fibroblasts.

### Downregulation of extracellular matrix-related proteins after HDAC inhibitor treatment

**Fig. 6:**
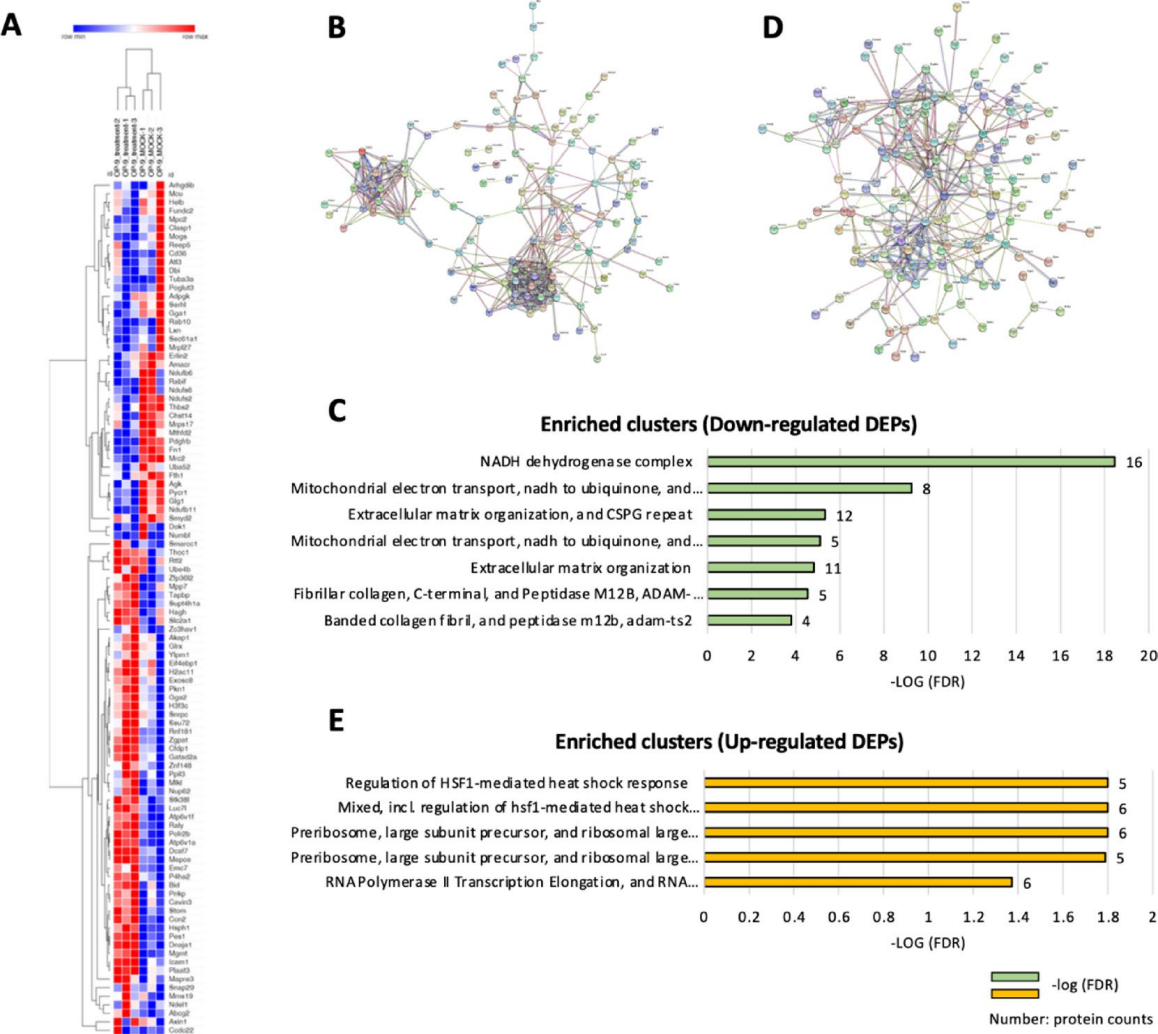
Protein expression profiling and PPI analysis and enriched clusters of DEPs from the proteomic analysis. PPI analysis using String networks reveals the tight interactions between the identified proteins. (**A**) A Heatmap of representative DEPs between OP-9 cells and J22352-treated OP-9 cells was generated using Morpheus networks by hierarchical clustering. The protein abundance is shown with colors from high (red) to low (blue). (**B**) PPI analysis of down-regulated DEPs and (**C**) the corresponding enriched clusters. (**D**) PPI analysis of up-regulated DEPs and (**E**) the related enriched clusters. PPI, protein–protein interaction; DEPs, differentially expressed proteins.

To further elucidate the potential drug mechanisms on the BM-derived myofibroblasts manipulated by J22352, J22352-treated OP-9 cells were subjected to LC-MS/MS analysis. The OP-9 cells were incubated with J22352 (12.5 µM) for 24 h, and the total proteins were extracted for analysis. The total protein profiles between J22352-treated OP-9 cells (treatment) and controls (MOCK) were analyzed. Herein, a total of 5154 proteins were identified, and 334 proteins were filtered as differentially expressed proteins (DEPs), including 187 up-regulated proteins and 147 down-regulated proteins. The differences of DEPs in the treated and MOCK cells demonstrated that the proteomic profiles were significantly changed with the J22352 incubation (Fig. 6A).

**Fig. 7:**
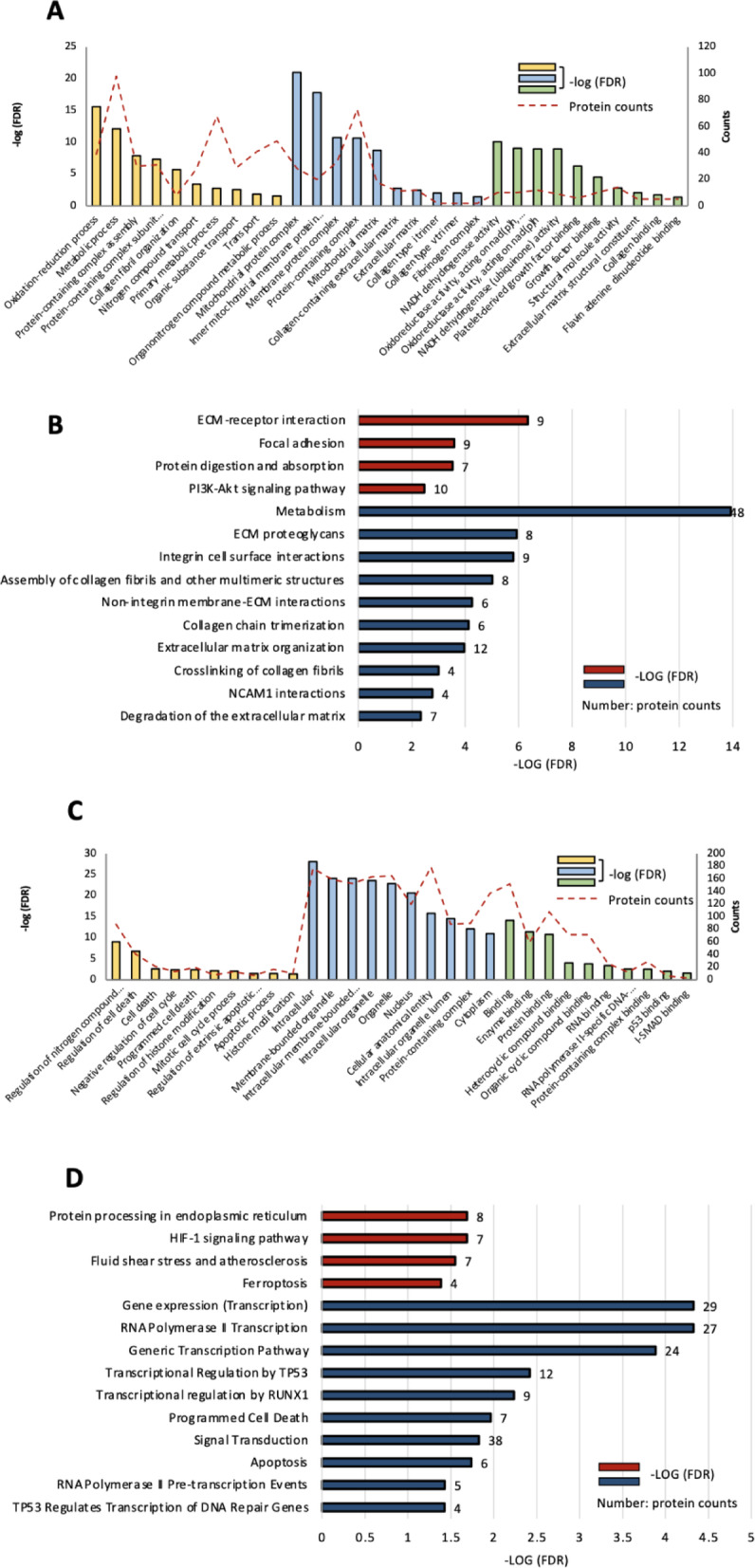
Enriched GO term, KEGG, and Reactome pathways involved with the DEPs. (**A**) Representative enriched GO terms, including BP, CC, and MF, and the (**B**) enriched KEGG and Reactome pathways in the down-regulated DEPs. (**C**) Representative enriched GO terms, including BP, CC, and MF, and the (**D**) enriched KEGG and Reactome pathways in the up-regulated DEPs. The color bar represents -log (FDR), and the red line and number beside the bars give the protein numbers involved in the certain signal pathway. GO, gene ontology; KEGG, Kyoto encyclopedia of genes and genomes; DEPs, differentially expressed proteins; BP, biological process; CC, cellular component; MF, molecular function; FDR, false detection rate.

To depict the interactions of these DEPs, the 187 up-regulated proteins and 147 down-regulated proteins were mapped to the protein–protein interactions (PPI) from the STRING database. The tight interaction among the down-regulated DEPs (Fig. 6B) indicated that the protein demonstrated highly similar biological functions and behaviors. The enriched clusters included the “NADH dehydrogenase,” “extracellular matrix organization,” and “fibrillar collagen” (Fig. 6C). Figure 6D shows the interaction among the up-regulated DEPs, and the enriched clusters were “heat shock factors,” “ribosomes,” and “RNA polymerases” (Fig. 6E). Functional analyses, including GO terms, KEGG, and Reactome pathways for DEPs, were separately performed between down-regulated and up-regulated DEPs. In the 147 down-regulated DEPs, most of the enriched GO terms were related to collagen formation and NADH dehydrogenase, including “collagen-containing extracellular matrix,” “NADH dehydrogenase activity,” and “protein-containing complex organization” (Fig. 7A). Enriched KEGG pathways were related to “ECM-receptor interaction,” “focal adhesion” and “protein digestion and absorption” (Fig. 7B, columns in red). Enriched Reactome pathways were correlated to “crosslinking of collagen fibrils” and “integrin cell surface interactions” (Fig. 7B, columns in red).

### Upregulation of cell programmed death pathways after HDAC inhibitor treatment

In the 187 up-regulated DEPs, most of the enriched GO terms were related to cell death and regulation of histone modification, including “apoptotic process,” “programmed cell death,” and “histone modification” (Fig. 7C). Enriched KEGG pathways were related to “ferroptosis,” and “HIF-1 signaling pathway” (Fig. 7D, columns in red). Enriched Reactome pathways were correlated to “programmed cell death,” “apoptosis,” and “RNA polymerase transcription” (Fig. 7D, columns in red). The proteomic analysis revealed that, after the treatment of a novel HDAC inhibitor, bone marrow myofibroblasts expressed a decreased level of collagen-related proteins. The apoptosis and ferroptosis effects were activated. These results support our hypothesis that the novel HDAC inhibitor reverses the collagen deposition in the bone marrow and alleviates organ fibrosis by triggering apoptosis and/or ferroptosis in the myofibroblasts.

## Discussion

In our previous study, we successfully synthesized an HDAC 6 inhibitor to combat pulmonary fibrosis; however, whether this drug can be applied to BMF needs to be further elucidated. Therefore, in the current study, we found that J22352 can effectively promote the apoptosis of bone marrow-derived fibroblasts and regulate the expression of fibrosis-related genes and proteins. In the proteomics results, after J22352 treatment, the total protein profiles significantly changed (Fig. 6). Many of the up-regulated DEPs are related to cell apoptosis, whereas the down-regulated DEPs are mostly involved in many biological pathways that promote fibrosis. Taken together, these findings indicate that J22352 has high potential as a candidate for treating BMF.

BM-derived fibroblasts exhibit increased proliferation mediated by TGF-β1, which induces fibroblast differentiation and ECM deposition, resulting in fibrosis (Fig. 1). These findings are consistent with previous research, highlighting the central role of TGF-β as the most potent and ubiquitous fibrogenic cytokine involved in fibrosis development across almost all organ systems^9–11^. Therefore, we established this platform to mimic the microenvironments in which the overproduction of TGF-β from megakaryocytes triggers fibrosis in the bone marrow^4^. Based on this platform, we found that J22352 exerted dose- and time-dependent anti-fibrotic effects (Fig. 3A), with J22352 demonstrating the most pronounced effects, achieved through the inhibition of cell growth and reduction of ECM deposition. To further analyze the anti-fibrotic and apoptosis-inducing mechanisms, we performed genetic and proteomic analyses. J22352 effectively inhibited the phosphorylation of Smad2/3 (Fig. 5). Smad2 and Smad3 are key proteins in the TGF-β signaling pathway that participate in the transduction of TGF-β signals from the cell membrane to the nucleus^9,23^. Once TGF-β binds to its receptor on the cell membrane, the receptor is then activated and phosphorylates Smad2/3 proteins^24^. Phosphorylated Smad2/3 forms a complex with Smad4, enters the nucleus, and participates in the regulation of gene transcription^25^. These findings might explain the mechanisms by which J22352 inhibits p-Smad2/3 expression by reducing HDAC6 activity, thereby blocking the TGF-β signaling pathway to reduce ECM deposition mediated by TGF-β and induce cell apoptosis.

Because of the complicated mechanisms of BMF, the only cure therapy for it is allogeneic hematopoietic stem cell transplantation^8^. Because Janus kinase 2 (Jak2) somatic mutations are identified in most of these patients, several targeted therapies against Jak2 have been proposed. Though these therapies provide some temporary relief of associated symptoms, they are not curative. In the case of ruxolitinib (a kind of Jak2 inhibitor), only the alleviation of constitutional symptoms was observed^26^. Furthermore, follow-up studies found that patients receiving ruxolitinib exhibited a reduced red blood cell count or anemia and thus reliance on blood transfusions^27^. These difficulties show that the current FDA-approved Jak inhibitors are still unable to effectively treat BMF. Therefore, there is still a need to develop molecular/targeted treatments other than Jak inhibition for BMF that are effective in eliminating other etiologies of this disease within the BM. For example, HDAC inhibition is a potent strategy to fight against this disease, for HDACs regulate gene expressions associated with TGF-β signaling and tissue fibrosis^12^. However, to the authors’ best knowledge, no HDAC inhibitor has been successfully translated into clinical use, and few studies illustrating HDAC inhibitors in BMF have been reported. Our study has demonstrated that the novel HDAC 6 inhibitor can alleviate the BMF by regulating fibrotic gene and/or protein expressions. These data show that this drug has a high potential to be applied to BMF in the future.

This study has some limitations. First, BMF might result from other diseases. This study provides clues that can effectively alleviate BMF, but developing novel treatments that can cure upstream diseases is needed. Second, the current in vitro study does not accurately mimic the actual physiological status in animals and/or humans. Further animal studies evaluating the efficacies and toxicity of the HDAC6 inhibitor are highly recommended.

In conclusion, the current study has demonstrated that J22352, an in-house synthesized HDAC6 inhibitor, inactivates the TGF-β signaling pathway, leading to reduced fibrosis and induction of cell death through apoptosis. To date, no HDAC inhibitors have been successfully translated to the clinic for treating BMF. This study gives insight into the therapeutic mechanisms of HDAC inhibitors and might provide hints for further studies.

## Materials and methods

### Cell culture and reagents

Murine bone marrow-derived fibroblast cell lines, M2-10B4 (number: 60228) and OP-9 (number: 60566), were purchased from Bioresource Collection and Research Center (BCRC, Hsinchu, Taiwan). M2-10B4 cells were cultured in RPMI-1640 medium (Thermo Fisher Scientific, Waltham, MA, USA) containing 10% heat-inactivated fetal bovine serum (FBS; Thermo Fisher Scientific) and 1% antibiotic-antimycotic (AA; CC501-0100, GeneDireX). OP-9 cells were grown in MEM alpha medium (12571048, Gibco) with 20% FBS and 1% AA. All cells were incubated at 37 °C, 95% humidity, and 5% CO_2_. Cells were routinely screened to prevent mycoplasma contamination (Sigma-Aldrich, St. Louis, MO, USA). The high-selective HDAC6 inhibitor J22352 was synthesized according to our previous study^28^.

### Cell viability

Cell viability was evaluated using the Cell Counting Kit-8 (CCK-8; C0005, TargetMol) according to the manufacturer’s instructions. Briefly, cells were seeded in 96-well flat-bottom microtiter plates at a density of 10,000 cells per well. After 24 h, the basal medium was substituted with fresh medium with TGF-β1 (10 ng/mL; Catalog No. 763104, BioLegend) for fibroblast differentiation^22^. Twenty-four hours later, J22352 was added at the concentrations indicated for 24, 48, and 72 h. After incubation, 10 µl (1:10 in volume) of CCK-8 reagent was added to the medium, and the cells were incubated for an additional 4 h. The cell viability was determined by measuring the absorbance at 450 and 690 nm with a microplate reader (SpectraMax^®^ M5 Microplate Reader, San Jose, CA, USA). The 50% inhibiting concentrations (IC_50_ values) of the drug were determined from dose-response curves.

### Determination of cell death

The APC Annexin V Apoptosis Detection Kit with PI (Biolegend, San Diego, CA, USA) was used to detect apoptosis according to the manufacturer’s instructions. Cells were seeded into a 10-cm dish. After attachment overnight, the cells were then stimulated with TGF-β1 for 24 h. After stimulation, cells were washed twice with PBS and incubated with a medium containing J22352 for the next 24 h. The same volume of DMSO (Sigma-Aldrich) without J22352 was added to the control group. All cells including the floating cells in the medium were harvested after incubation. Cells were collected, rinsed twice with PBS, and resuspended in Annexin V binding buffer at a concentration of 10^6^ cells/mL. A total of 100 µL of cell suspension was mixed with 5 µL of Annexin V-APC and incubated for 15 min at room temperature in the dark. Then 400 µL of binding buffer and 2.5 µL propidium iodide (PI) were added. The mixture was incubated for 5 min at room temperature in the dark and then analyzed by an LSR Fortessa Flow Cytometer (BD Biosciences, San Jose, CA, USA). Data were analyzed in FlowJo v10 software (BD Biosciences).

### RNA extraction, cDNA synthesis, and qualitative PCR

The TRIzol reagent (Invitrogen Life Technologies, USA) was utilized to extract RNA according to the manufacturer’s instructions. Briefly, after being harvested and washed, the cells were resuspended in TRIzol reagent and incubated for 10 min at 4 °C. Following chloroform extraction, RNA was precipitated with cold isopropanol. The sample was then subjected to centrifugation at 10,000 g for 15 min and the precipitated pellet was obtained, washed with 70% ethanol, and dried in a vacuum chamber. The RNA was dissolved in diethylpyrocarbonate-treated water (DEPC-water) and measured with a Nano photometer™ (Implen GmbH, Munich, Germany). Subsequently, a mixture containing a total of 1 µg RNA, 1 µl of 50 µM Oligo dT primers (Sigma-Aldrich), 1 µl of 50 nM random primers (Sigma-Aldrich), 1 µl of dNTP, and a volume of Ulter Pure DEPC Water (Protech, Taipei, Taiwan) were incubated at 65 °C for 5 min following a 10-minute incubation at 4 °C. A total of 4 µl of 5x first strand buffer (Invitrogen), 2 µl of 100 mM DTT (Invitrogen), 1 µl of RNase-free water, and 1 µl of SuperScript II RT (Invitrogen) were added to the denatured RNA. The RNA solution was incubated at 50 °C for 60 min and 75 °C for 15 min, respectively.

Quantitative PCR was performed (in triplicate) using SYBR Green PCR Master Mix (Bio-Rad, California, USA) according to the manufacturer’s instructions in a qPCR machine (Bio-Rad). The primer sequences are listed in Table S1. The relative amounts of mRNA in each sample were calculated based on its threshold cycle compared with the threshold cycle of the housekeeping gene, β-actin. The results were presented as 2^−(Ct of target gene – Ct of housekeeping gene),^ (2^− DCT^) in arbitrary units and analyzed in IQ5 analysis software (Bio-Rad, USA).

### Western blot

Cells were harvested at 80% confluence and lysed with radioimmunoprecipitation assay (RIPA) buffer (Sigma–Aldrich, Catalog No. R0278) supplemented with protease inhibitors (Thermo Fisher Scientific, Catalog No. 78425) and phosphatase inhibitors (Thermo Fisher Scientific, Catalog No. 78420). After 30 min of incubation at 4 °C, the proteins were collected by centrifugation at 16,000 g for 20 min. The protein concentration was determined using a BCA Protein Assay Kit (Bio-Rad, Hercules, CA, USA). Extracted proteins (30 µg) were denatured by boiling in a sample buffer at 95 °C for 10 min. Samples were then separated by electrophoresis into an SDS-page gel and transferred to a PVDF membrane. The membrane was blocked with 5% skim milk for 1 h at room temperature. Next, the membranes were incubated at 4 °C overnight with specific primary antibodies (Table S2). After three washes for 30 min with TBST (0.05% Tween-20 in TBS), membranes were incubated with a household peroxidase (HRP)-conjugated secondary antibody for 1 h at room temperature and then washed three times with TBST. Bands were detected using the ECL prime kit (Bio-Rad Laboratories, Hercules, CA, USA). All the original blots were provided in Figure S1 to S7.

### HDAC6 activity assay

The HDAC6 activity assay (Abcam, Catalog No. ab284549) was conducted according to the manufacturer’s instructions. Briefly, 1 × 10^6^ cells were mixed with 100 µL HDAC6 lysis buffer for 5 min on ice, followed by centrifugation at 16,000 g for 10 min. The supernatants were collected and placed on ice and the amounts of protein were quantified by a BCA Protein Assay Kit (Bio-Rad). Then, for each sample, 2 µL of lysate was added to a white 96-well plate. For inhibitor control, a parallel sample contained the same amount of lysate and an additional 2 µL of a 10-fold diluted HDAC6 inhibitor (in DMSO). The mixtures were incubated at 37 °C for 10 min. As a positive control, 2 µLof HDAC6 positive control was diluted with 500 µl of HDAC6 assay buffer. A total of 25 µL of positive control mixture was added to the wells. Then the volumes of samples and positive control were adjusted to 50 µL per well with HDAC6 assay buffer. A total of 50 µL of substrate mix solution was added to each well, followed by a 30-minute incubation at 37 °C. After incubation, 10 µL of developer was added to stop the reactions. The plates were gently mixed and incubated at 37 °C for 10 min to generate fluorescence. A microplate reader (SpectraMax^®^ M5 Microplate Reader, San Jose, CA, USA) was used to determine the fluorescence signal at Ex/Em 380/490 nm in an endpoint mode at 37 °C. The HDAC6 activities were quantified by interpolation from the standard curve generated from the raw values of the provided calibrators.

### LC-MS/MS

The protein extracts were precipitated according to a previous study^29^. Briefly, a 4-fold volume of pre-chilled acetone was added to the protein samples and incubated for 1 h at -80 °C. After centrifugation at 14,000 g for 10 min, the precipitated proteins were washed twice with 80% cold acetone. The resulting pellets were resuspended in 6 M urea and total protein concentration was determined with a BCA kit (Bio-rad). A total of 50 µg proteins were reduced by 5 mM dithiothreitol (DTT) at 29 °C for 45 min. The reduced proteins were then alkylated with 10 mM iodoacetamide in the dark at 29 °C for 45 min. Next, trypsin (Promega, Madison, WI, USA; V5111) with a 50:1 (w/w) ratio was added for protein digestion at 29 °C for 16 h. The reaction was inhibited by adding 10% trifluoroacetic acid (TFA) to the final concentration of 0.5% TFA. The final solution was desalted using the C_18_ procedure.

Liquid chromatography-tandem mass spectrometry (LC-MS/MS) was performed using an Orbitrap Fusion Lumos Tribrid quadrupole-ion trap-Orbitrap mass spectrometer (Thermo Fisher Scientific). The peptides were separated through a C_18_ Acclaim PepMap NanoLC column (2.0 μm × 75 μm × 250 mm) (Thermo Fisher Scientific) coupled with an Ultimate System 3000 nanoLC system (Thermo Fisher Scientific). The peptides were carried by a liquid consisting of two components: mobile phase A with 0.1% formic acid (FA) in H_2_O and mobile phase B with 0.1% FA in acetonitrile (CAN). Peptides were released at a rate of 300 ng/mL, with a gradient of 2–40% over a period of 90 min. The analysis was conducted using a full MS scan, followed by HCD-MS/MS of the most intense ions within 3 s. The goal of mass accuracy calibration was to achieve an accuracy of less than 5 ppm. The resolution for MS1 was set at 120,000 (m/z 200), and a target ion value of 5e5 was selected for both scans. The maximum ion injection time for the full scan was 50 msec. For HCD-MS/MS fragmentation, a resolution of 15,000 was used with a 1.4 Da isolation window and a normalized collision energy of 32. During MS/MS analysis, ions previously selected were excluded for 180 s. The AGC target was set to 5e4, which was 50 msec. Previously selected ions were also excluded for 180 s. The MS/MS spectra were searched against a mouse sequence database comprising 17,033 entries (released in March 2020) using the Mascot search engine (Matrix Science, London, UK; version 2.3) through Proteome Discoverer 2.2. The label-free quantification parameters included trypsin digestion allowing for up to two missed cleavages. The precursor mass tolerance was set to 10 ppm, and the fragment mass error tolerance was set to 0.02 Da. The protein analysis involved modifications such as deamidation (NQ) and oxidation (M), with carbamidomethylation (C) remaining constant. To minimize false positives, peptide mass tolerance was utilized with a false detection rate (FDR) that was less than 1%. Differentially expressed proteins (DEPs) were identified by determining the proteins with log_2_ ratios above the mean of total log_2_ ratios plus one standard deviation (SD) as over-expressed, while those with log_2_ ratios below par minus one SD were considered down-expressed. The abundances of all the DEPs were summarized in Table S3. The MS proteomics data have been deposited to the ProteomeXchange Consortium via the PRIDE^30^ partner repository with the dataset identifier PXD055276.

### Bioinformatic analysis

Identified DEPs were subjected to Database Annotation Visualization and Integrated Discovery (DAVID, http://david.abcc.ncifcrf.gov/) to investigate the Gene ontology (GO) terms, including biological processes (BP), cellular components (CC), and molecular functions (MF)^31^ and enriched Kyoto Encyclopedia of Genes and Genomes (KEGG) pathways^32^. The enriched biomolecular pathways in which DFPs are involved were generated by the Reactome (http://reactome.org)^33^. The clustered heat map was generated by Morpheus (https://software.broadinstitute.org/morpheus/)^34^ to compare the expression patterns of DEPs. The Search Tool for the Retrieval of Interacting Genes/Proteins (STRING, https://string-db.org/) network was employed to establish a protein–protein interaction (PPI) network of DEPs^35^.

### Statistical analysis

The data are presented as mean ± SEM. The experiments were conducted at least three times, and consistent results were achieved. The Shapiro-Wilk test was employed to determine whether the data showed a normal distribution. Statistical analysis was conducted using the Student’s *t*-test or the Mann-Whitney test in GraphPad Prism software 9.0. A *p*-value < 0.05 was considered statistically significant.

## Electronic supplementary material

Below is the link to the electronic supplementary material.

Supplementary Material 1.

Supplementary Material 2.

Supplementary Material 3

## Data Availability

The data presented in this study are available on request from the corresponding author and/or are available via ProteomeXchange with identifier PXD055276.
