# Supplementary material for: High-selective HDAC6 inhibitor alleviates bone marrow fibrosis through inhibiting collagen formation and extracellular matrix deposition
Source: Sci Rep. 2025 Aug 1;15:28105. doi: 10.1038/s41598-025-08384-6 (PMC12317122; doi:10.1038/s41598-025-08384-6)
Supplement: Supplementary file 1 — Supplementary Material 1. [file 41598_2025_8384_MOESM1_ESM.docx]

**Table S1.** Primer sequences used in this study.

| **Gene** | **Forward** | **Reverse** | **Reference** |
| --- | --- | --- | --- |
| COL1A1 | GAGGTCCTGGTGAAGTTGGT | CAGGGAAGCCTCTTTCTCCT | [1] |
| α-SMA | CCACCGCAAATGCTTCTAAGT | GGCAGGAATGATTTGGAAAGG | [2] |
| COL3A1 | TAAAGAAGTCTCTGAAGCTGATGG | ATCTATGATGGGTAGTCTCATTGC | [3] |
| CTGF | TCCCGAGAAGGGTCAAGCT | TCCTTGGGCTCG-TCACACA | [4] |
| Periostin | CAGAGTTCATGTCTTTGTTTCTGC | CTGTGCAAAGACTTAGCTGTATG | [3] |
| Elastin | CAGTCGCTTCACCTACAGCA | CGGGAGGTCTTGGTGGTTTT | [5] |
| MMP-9 | CCACATCTCCCTCCAGAAA | CACTTGGTGGTTTGCTACGA | [6] |
| Fibronectin | TGACTGGCCTTACCAGAGGG | CATCTGTAGGCTGGTTCAGGC | [7] |
| β-actin | ATCTGGCACCACACCTTCTACAATG | CACGCTCGGTCAGGATCTTCATG | [8] |

**Table S2.** Antibody information of Western blot used in this study.

| **Antibody** | **Supplier** | **Cat. No.** | **City** | **Country** |
| --- | --- | --- | --- | --- |
| COL1A1 | Elabscience | E-AB-70008 | Wuhan | China |
| Alpha-SMA | Elabscience | E-AB-34268 | Wuhan | China |
| Histone H3 | Elabscience | E-AB-31686 | Wuhan | China |
| Ac-H3 | Elabscience | E-AB-67839 | Wuhan | China |
| GAPDH | Elabscience | E-AB-20072 | Wuhan | China |
| Caspase-3 | Cell signaling | 14200 | Danvers, Massachusetts | USA |
| Cleaved caspase-3 | Cell signaling | 9664 | Danvers, Massachusetts | USA |
| Cleaved PARP | Cell signaling | 9548 | Danvers, Massachusetts | USA |
| COL3A1 | Abcam | ab7778 | Cambridge | UK |
| Elastin | Abcam | ab217356 | Cambridge | UK |
| CTGF | Abcam | ab6992 | Cambridge | UK |
| Periostin | Abcam | ab92460 | Cambridge | UK |
| p-Smad2/3 | Abcam | ab272332 | Cambridge | UK |
| Smad2/ 3 | Abcam | ab202445 | Cambridge | UK |

**Reference**

1. Adapala, V.J.; Adedokun, S.A.; Considine, R.V.; Ajuwon, K.M. Acute inflammation plays a limited role in the regulation of adipose tissue COL1A1 protein abundance. *The Journal of nutritional biochemistry* **2012**, *23*, 567-572.

2. Li, Y.H.; Woo, S.H.; Choi, D.H.; Cho, E.-H. Succinate causes α-SMA production through GPR91 activation in hepatic stellate cells. *Biochemical and biophysical research communications* **2015**, *463*, 853-858.

3. Hara, M.; Yokota, K.; Saito, T.; Kobayakawa, K.; Kijima, K.; Yoshizaki, S.; Okazaki, K.; Yoshida, S.; Matsumoto, Y.; Harimaya, K. Periostin promotes fibroblast migration and inhibits muscle repair after skeletal muscle injury. *JBJS* **2018**, *100*, e108.

4. Bonniaud, P.; Martin, G.; Margetts, P.J.; Ask, K.; Robertson, J.; Gauldie, J.; Kolb, M. Connective tissue growth factor is crucial to inducing a profibrotic environment in “fibrosis-resistant” BALB/c mouse lungs. *American journal of respiratory cell and molecular biology* **2004**, *31*, 510-516.

5. Chen, W.; Yan, X.; Xu, A.; Sun, Y.; Wang, B.; Huang, T.; Wang, H.; Cong, M.; Wang, P.; Yang, A. Dynamics of elastin in liver fibrosis: accumulates late during progression and degrades slowly in regression. *Journal of cellular physiology* **2019**, *234*, 22613-22622.

6. Murthy, S.; Ryan, A.; He, C.; Mallampalli, R.K.; Carter, A.B. Rac1-mediated mitochondrial H2O2 generation regulates MMP-9 gene expression in macrophages via inhibition of SP-1 and AP-1. *J Biol Chem* **2010**, *285*, 25062-25073, doi:10.1074/jbc.M109.099655.

7. Roman, W.; Martins, J.P.; Gomes, E.R. Local Arrangement of Fibronectin by Myofibroblasts Governs Peripheral Nuclear Positioning in Muscle Cells. *Developmental Cell* **2018**, *46*, 102-111.e106, doi:<https://doi.org/10.1016/j.devcel.2018.05.031>.

8. Lin, G.; Yu, Q.; Xu, L.; Huang, Z.; Mai, L.; Jiang, L.; Su, Z.; Xie, J.; Li, Y.; Liu, Y. Berberrubine attenuates potassium oxonate-and hypoxanthine-induced hyperuricemia by regulating urate transporters and JAK2/STAT3 signaling pathway. *European Journal of Pharmacology* **2021**, *912*, 174592.
