# Supplementary material for: High-selective HDAC6 inhibitor alleviates bone marrow fibrosis through inhibiting collagen formation and extracellular matrix deposition
Source: Sci Rep. 2025 Aug 1;15:28105. doi: 10.1038/s41598-025-08384-6 (PMC12317122; doi:10.1038/s41598-025-08384-6)

# Supplementary Figure 1. Original blots of Figure 1C.

**α-SMA (M2-10B4)**

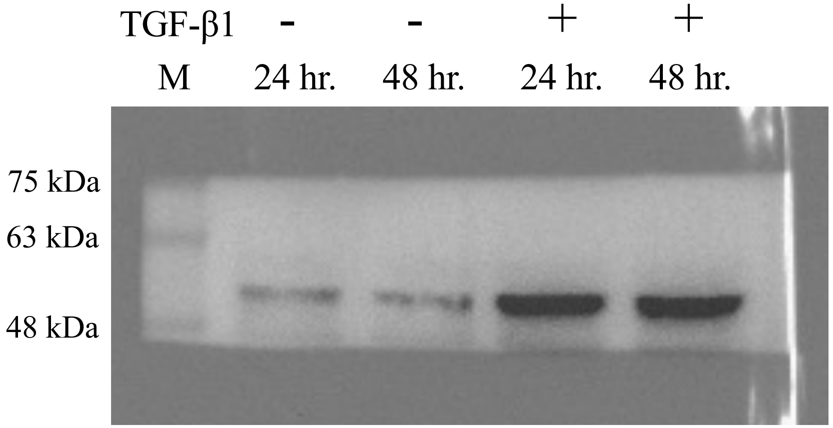


**GAPDH (M2-10B4)**

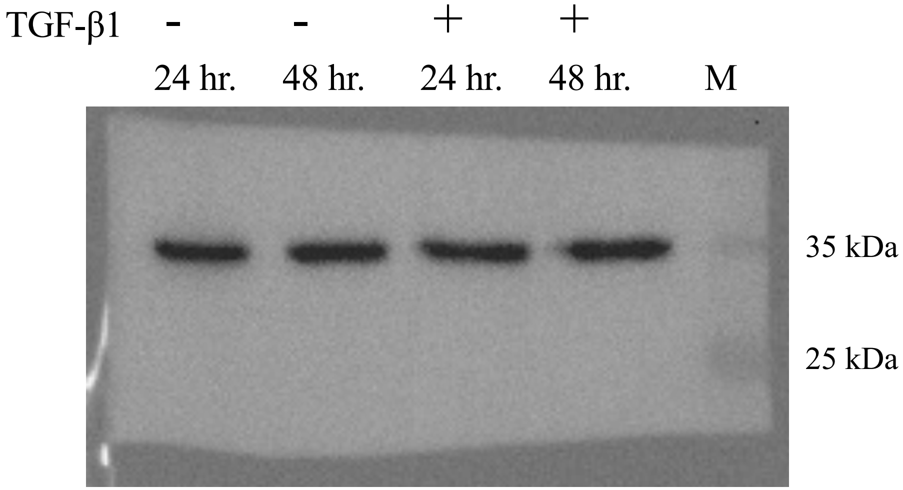


**COL1A1 (M2-10B4)**

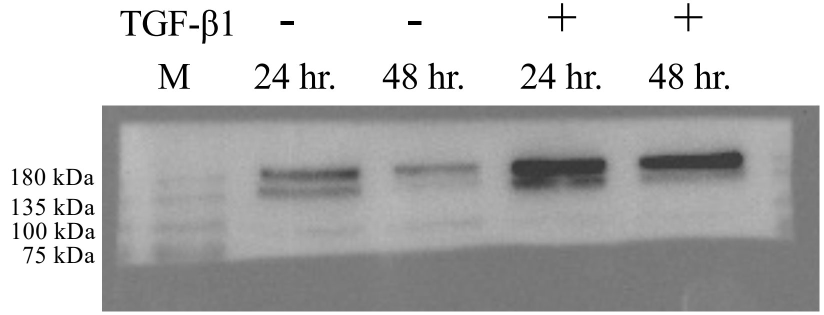

# Supplementary Figure 2. Original blots of Figure 1D.

**α-SMA (OP-9)**

TGF-β1 - - + +

M 24 hr. 48 hr. 24 hr. 48 hr.

75 kDa
63 kDa
48 kDa

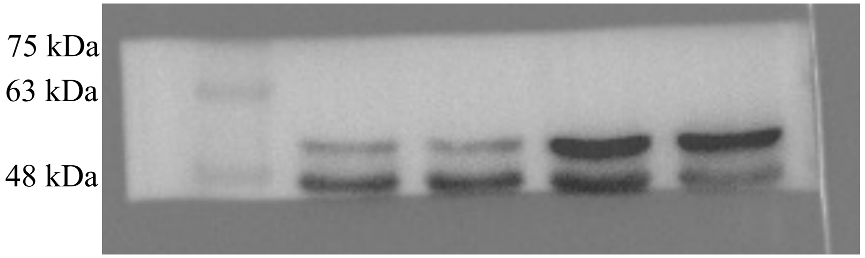

**GAPDH (OP-9)**

TGF-β1 - - + +

24 hr. 48 hr. 24 hr. 48 hr. M

35 kDa
25 kDa

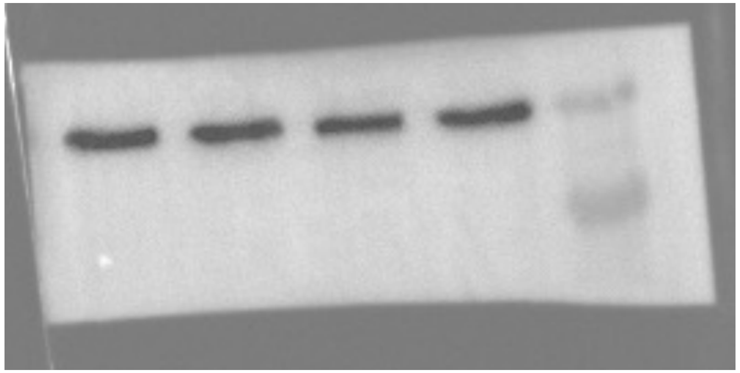

**COL1A1 (OP-9)**

TGF-β1 - - + +

M 24 hr. 48 hr. 24 hr. 48 hr.

180 kDa
135 kDa
100 kDa
75 kDa
63 kDa

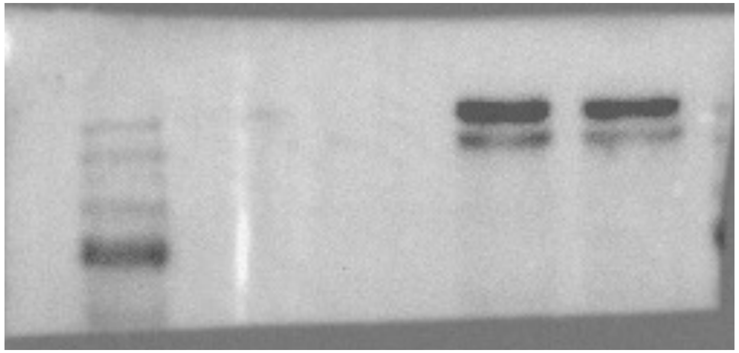

# Supplementary Figure 3. Original blots of Figure 2A (M2-10B4).

**H3K27ac (M2-10B4)**

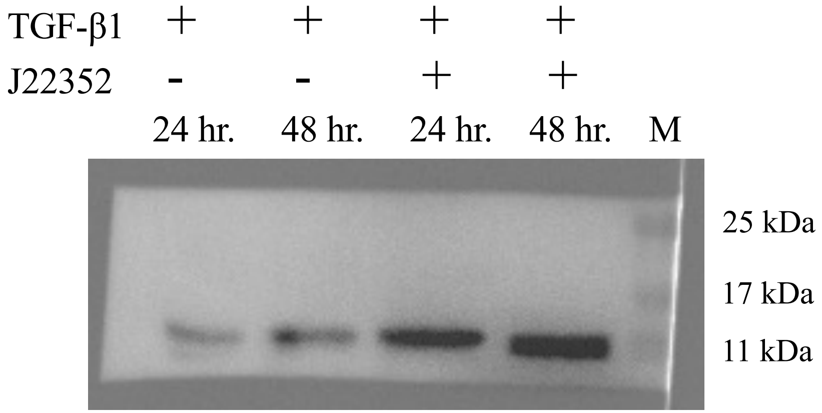


**H3 (M2-10B4)**

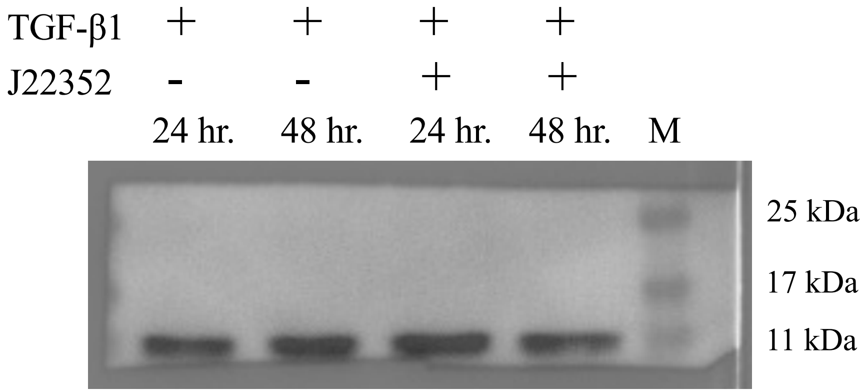


**GAPDH (M2-10B4)**

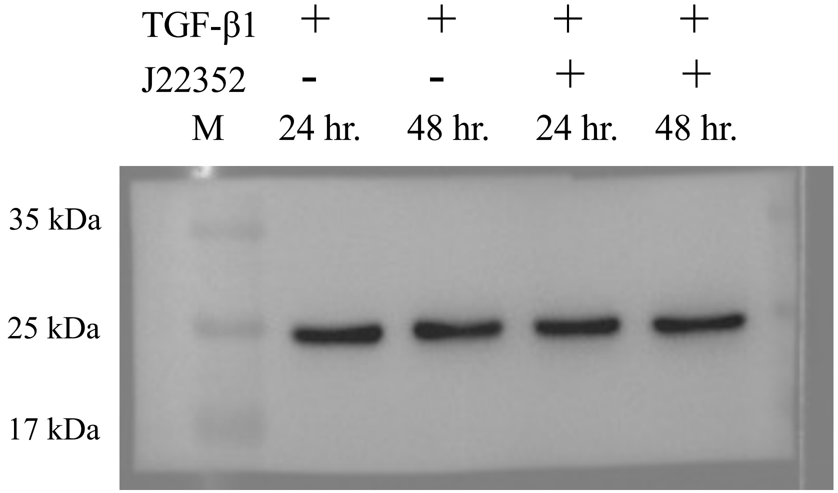

# Supplementary Figure 4. Original blots of Figure 2A (OP-9).

### H3K27ac (OP-9)

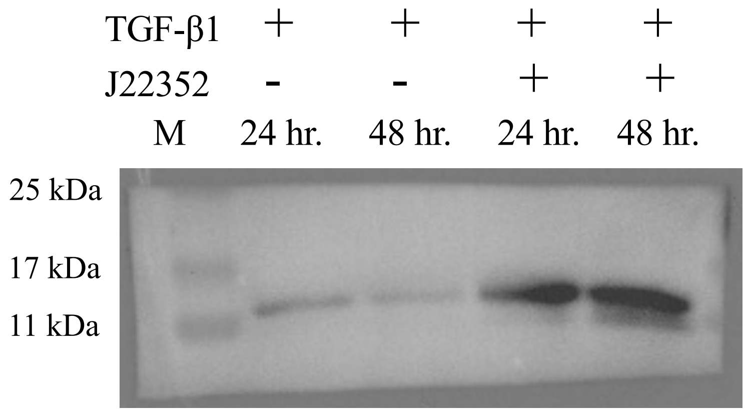


### GAPDH (OP-9)

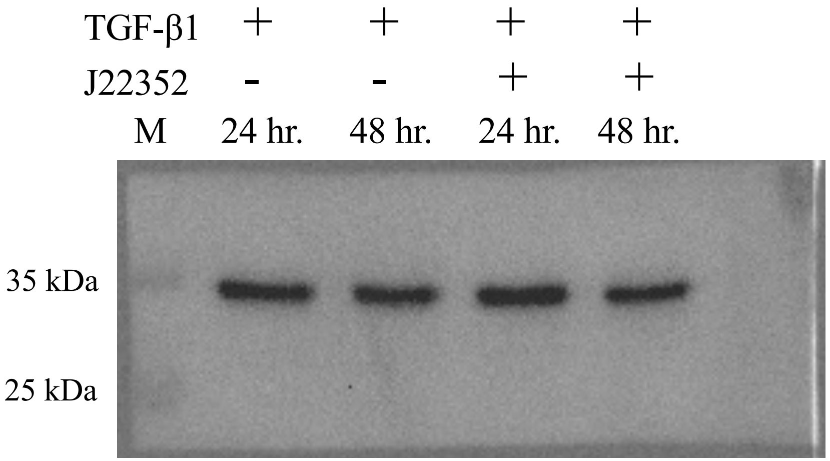


### H3 (OP-9)

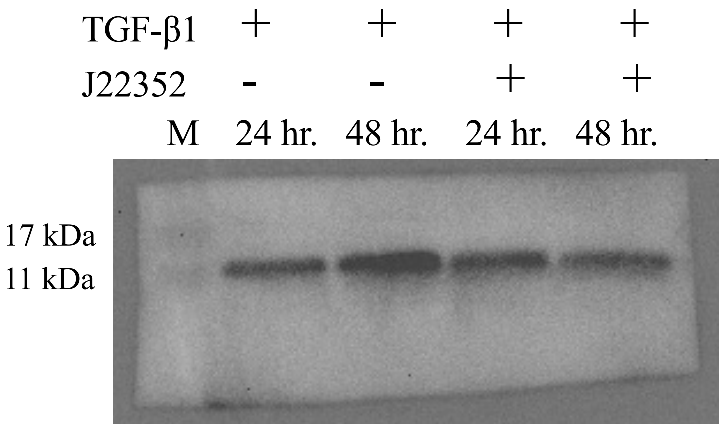

## Supplementary Figure 5. Original blots of Figure 3D.

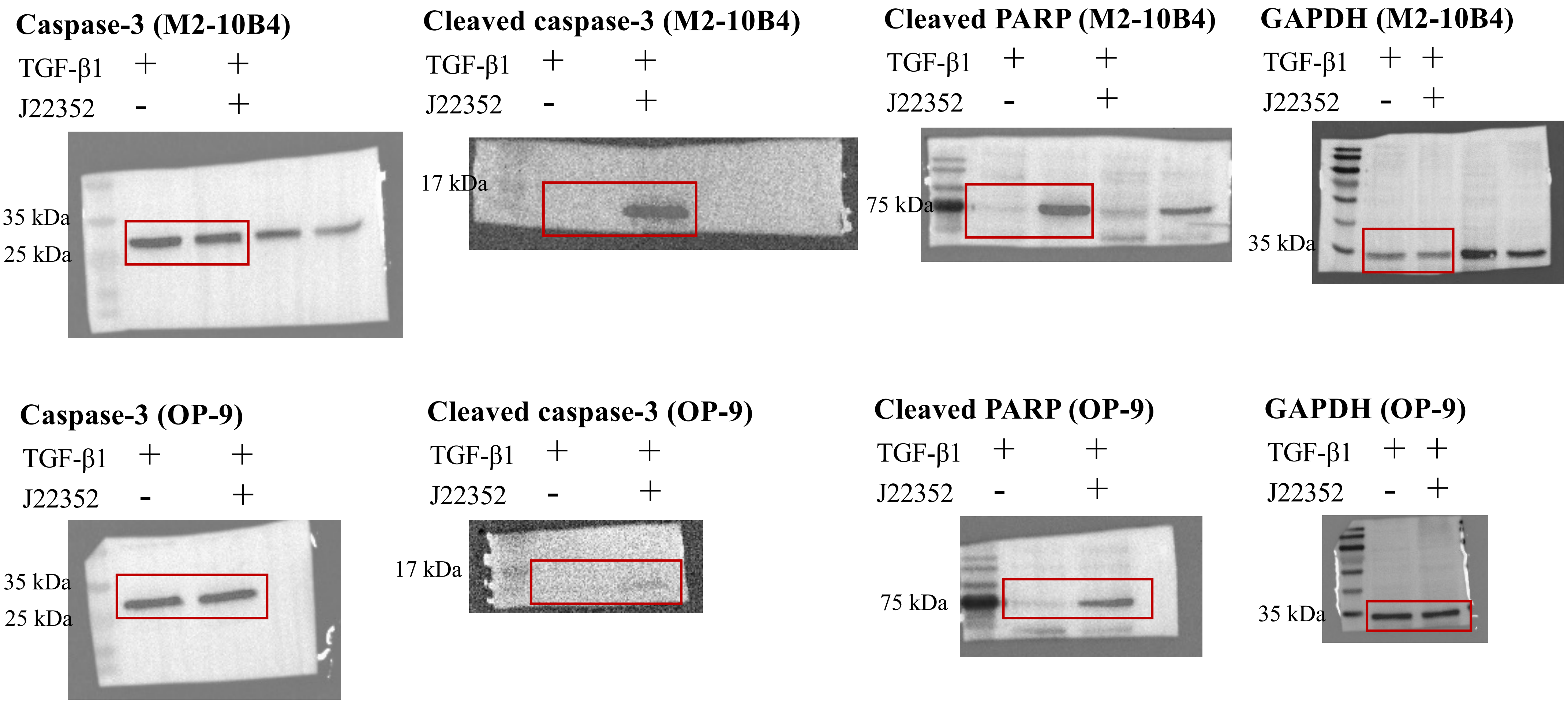

## Supplementary Figure 6. Original blots of Figure 5A (M2-10B4).

**α-SMA (M2-10B4)**

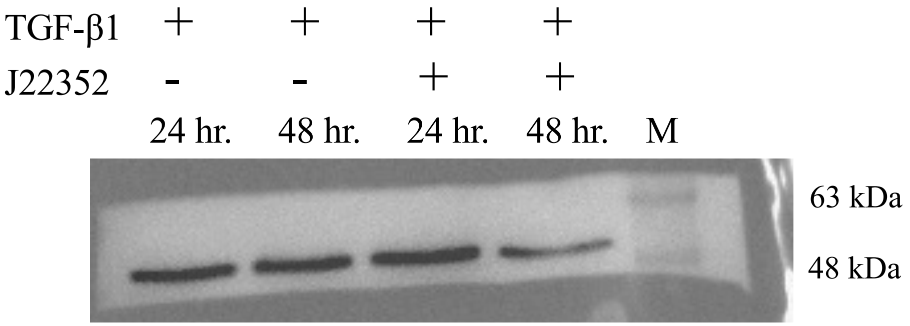


**COL1A1 (M2-10B4)**

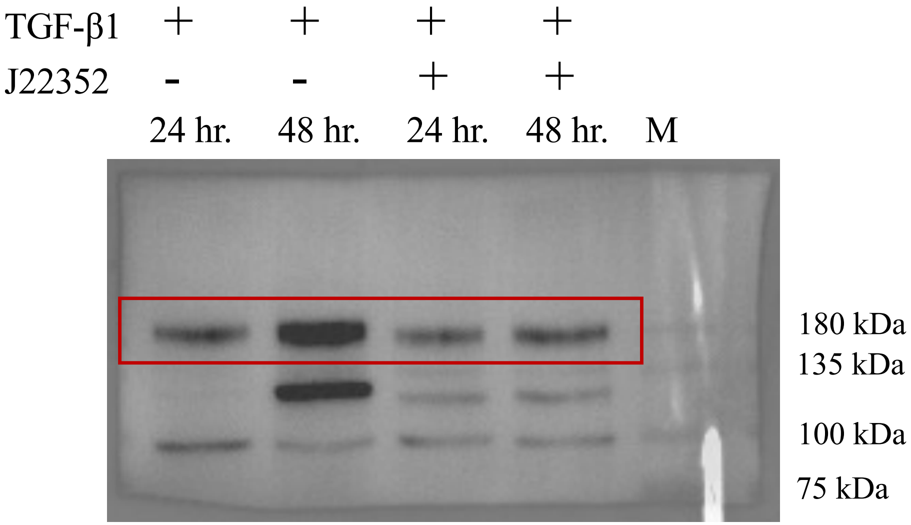


**COL3A1 (M2-10B4)**

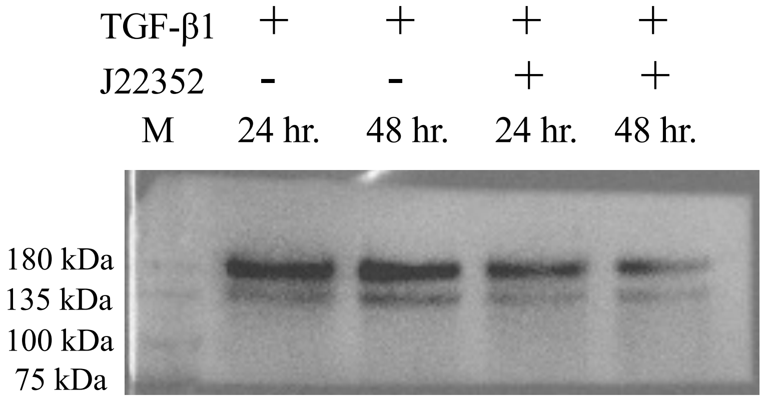


**CTGF (M2-10B4)**

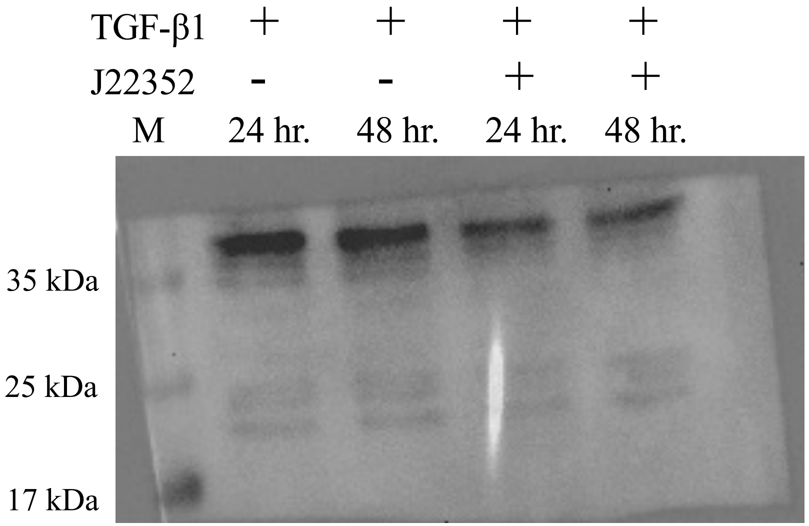

# Supplementary Figure 6. Original blots of Figure 5A (M2-10B4)-continue.

**Elastin (M2-10B4)**

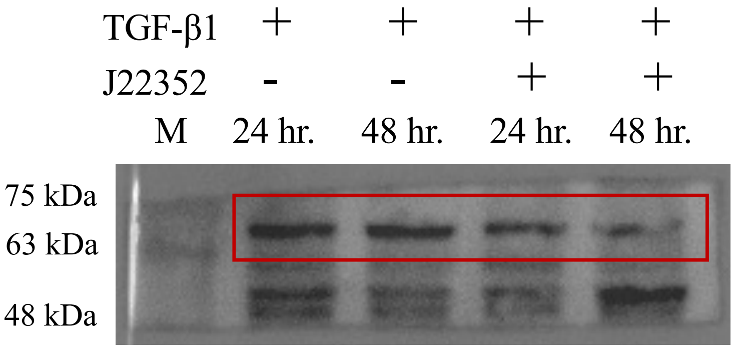


**Periostin (M2-10B4)**

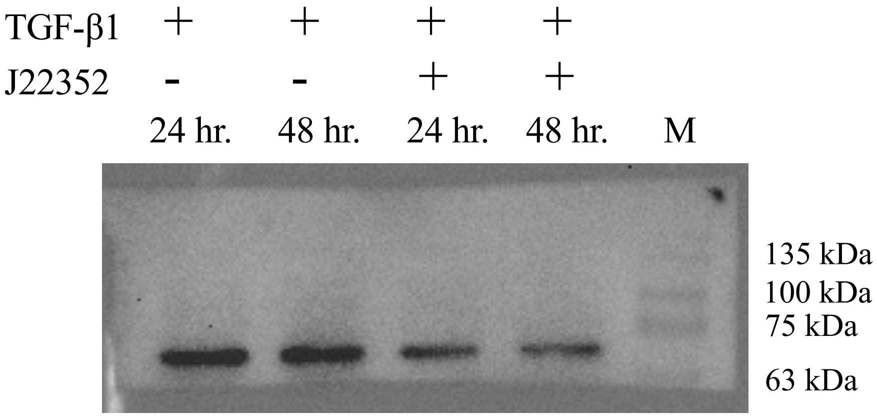


**Smad2/3 (M2-10B4)**

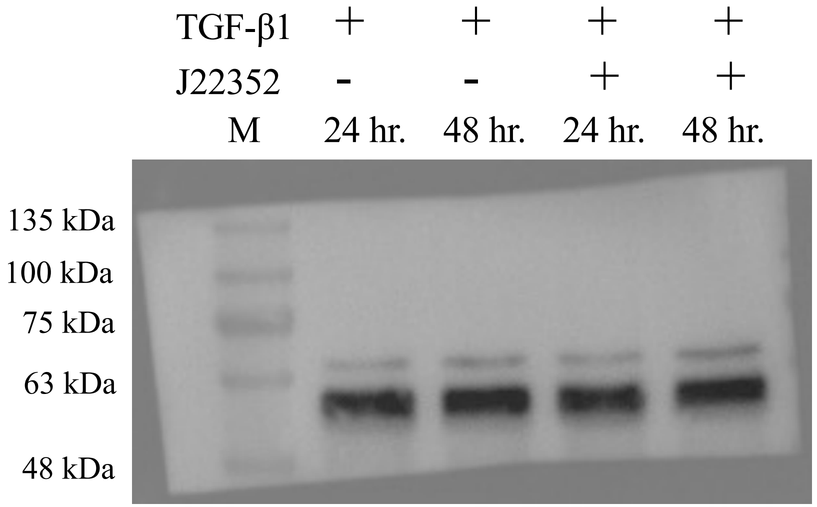

## Supplementary Figure 6. Original blots of Figure 5A (M2-10B4)-continue.

**P-Smad2/3 (M2-10B4)**

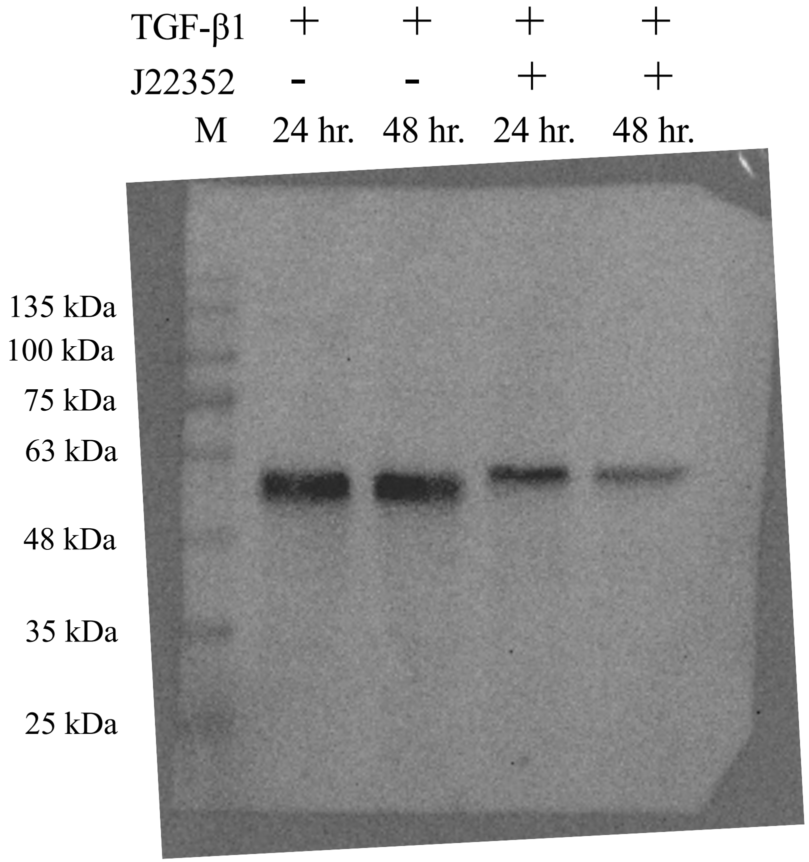


**GAPDH (M2-10B4)**

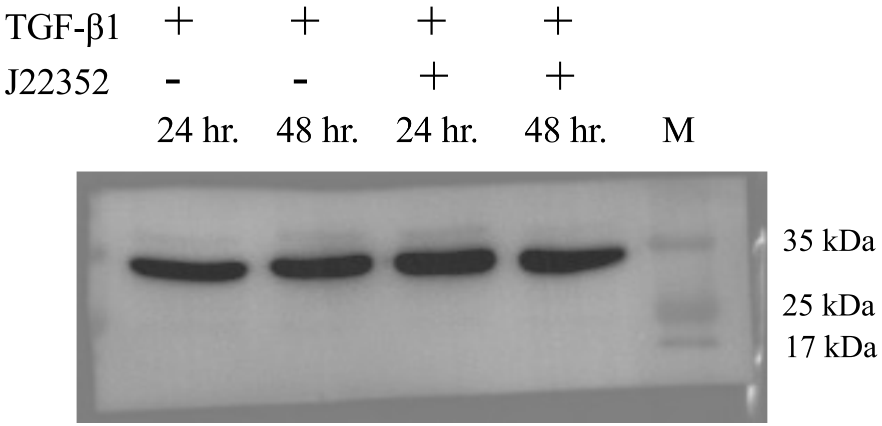

# Supplementary Figure 7. Original blots of Figure 5A (OP-9).

**α-SMA (OP-9)**

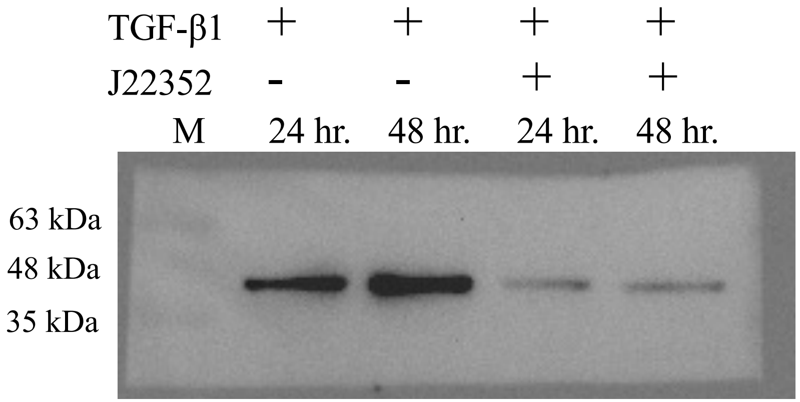


**COL1A1 (OP-9)**

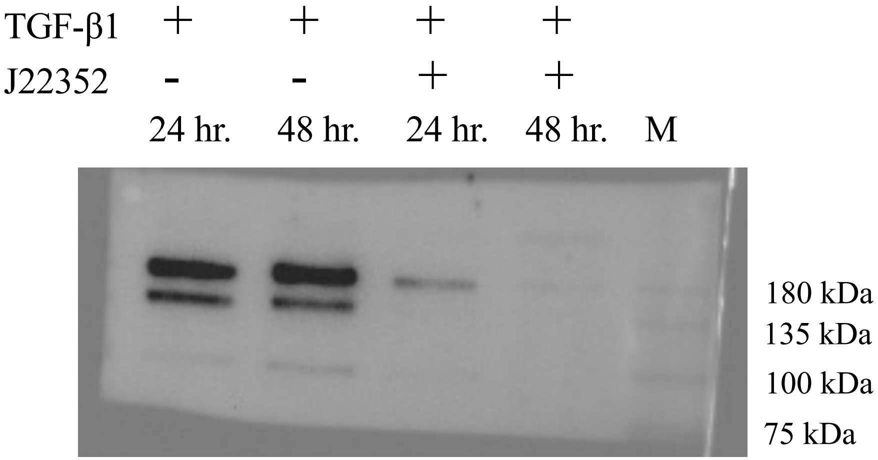


**COL3A1 (OP-9)**

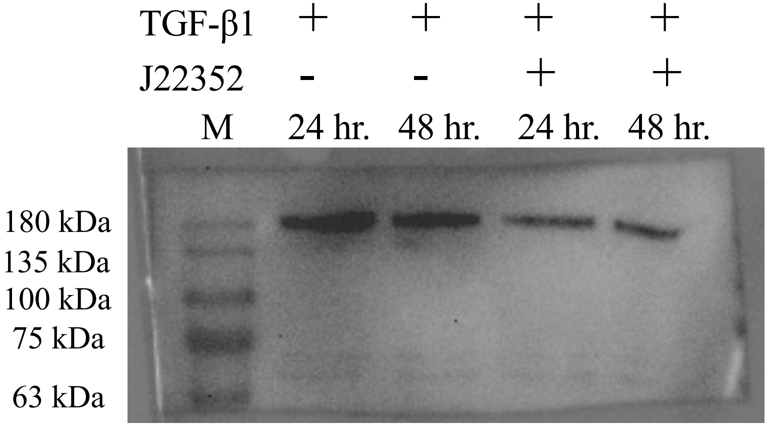


**CTGF (OP-9)**

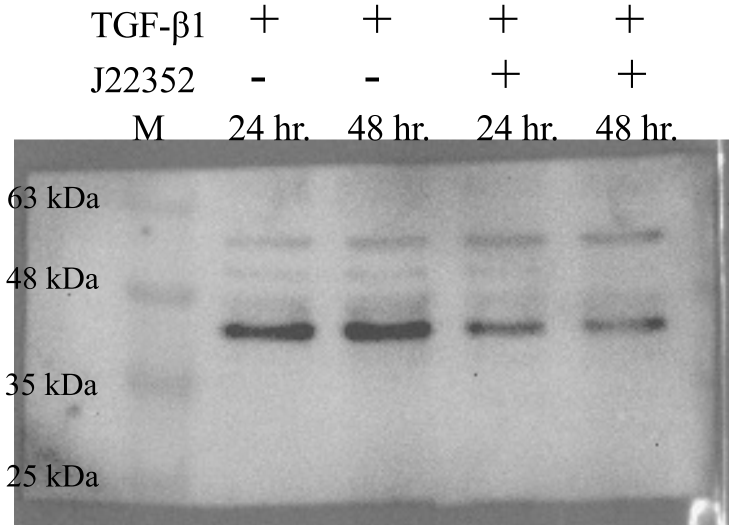

# Supplementary Figure 7. Original blots of Figure 5A (OP-9)-continue.

**Elastin (OP-9)**

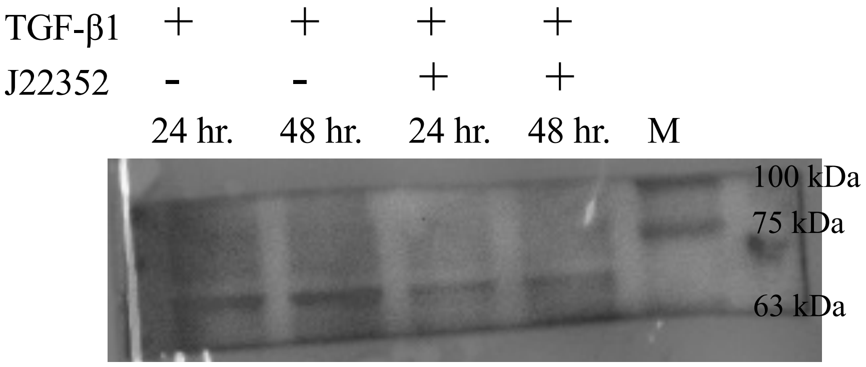


**Periostin (OP-9)**

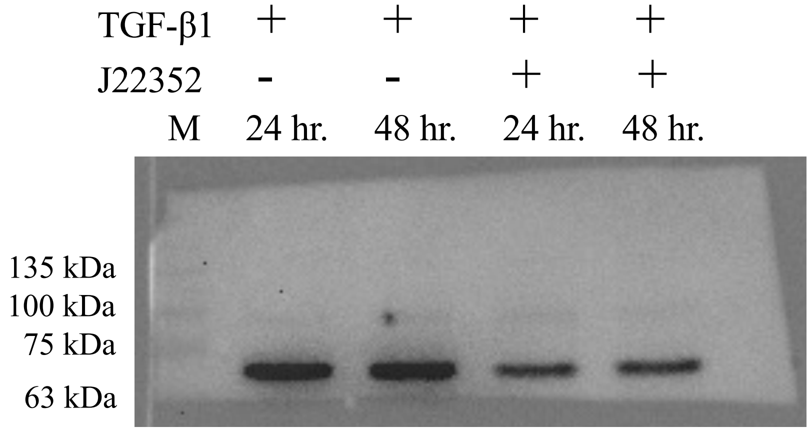


**Smad2/3 (OP-9)**

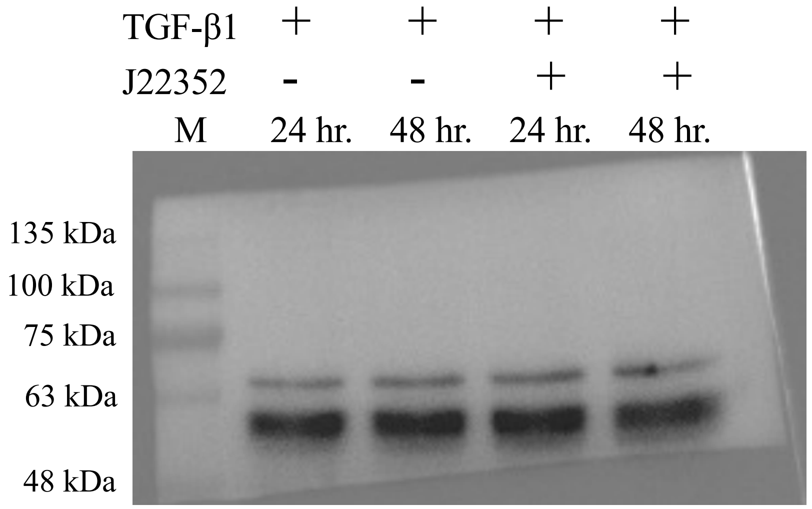

## Supplementary Figure 7. Original blots of Figure 5A (OP-9)-continue.

**P-Smad2/3 (OP-9)**

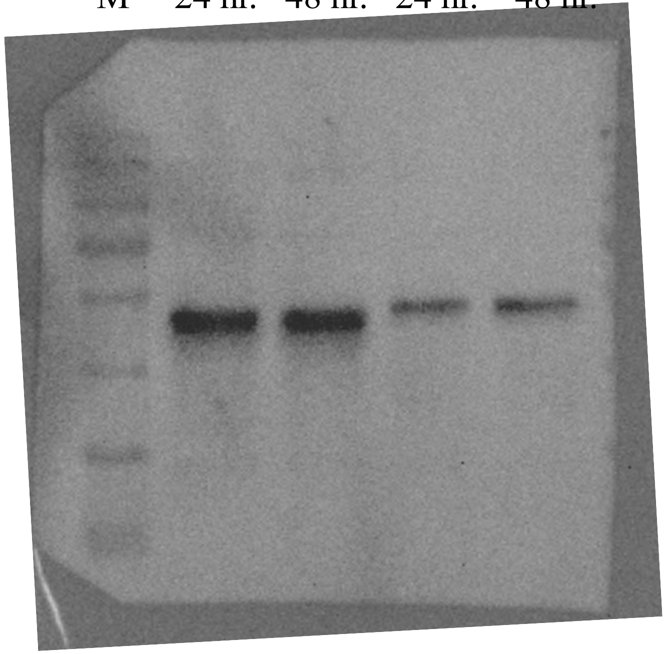


**GAPDH (OP-9)**

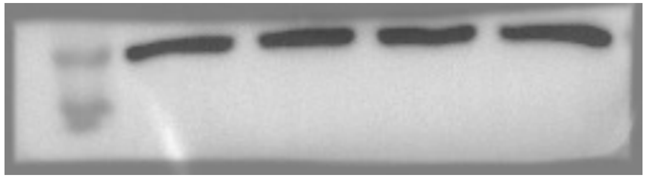

Supplement: Supplementary file 3 — Supplementary Material 3 [file 41598_2025_8384_MOESM3_ESM.pdf]
